# Environmental factors shaping the diversity of bacterial communities that promote rice production

**DOI:** 10.1186/s12866-018-1174-z

**Published:** 2018-06-04

**Authors:** Zhaohui Wu, Qingshu Liu, Zhenyu Li, Wei Cheng, Jimin Sun, Zhaohui Guo, Yongmei Li, Jianqun Zhou, Delong Meng, Hongbo Li, Ping Lei, Huaqun Yin

**Affiliations:** 1 Hunan Hybrid Rice Research Center/State Key Laboratory of Hybrid Rice, Changsha, 410125 China; 2 Hunan Institute of Microbiology, Changsha, 410009 China; 3 College of Plant Protection, Hunan Agricultural University, Changsha, 410128 China; 4 School of Minerals Processing and Bioengineering, Central South University, Changsha, 410083 China; 5 Hunan Soil and Fertilizer Institute, Changsha, 410125 China; 6 Hunan Institute of Agricultural Information and Engineering, Changsha, 410125 China; 7 LongPing Graduate Institute, Hunan University, Changsha, 410125 China

**Keywords:** Bacterial diversity, Bacterial community structure, Super hybrid rice, 16S rRNA pyrosequencing technology, Crop yield, Soil physicochemical properties

## Abstract

**Background:**

Exploiting soil microorganisms in the rhizosphere of plants can significantly improve agricultural productivity; however, the mechanism by which microorganisms specifically affect agricultural productivity is poorly understood. To clarify this uncertainly, the rhizospheric microbial communities of super rice plants at various growth stages were analysed using 16S rRNA high-throughput gene sequencing; microbial communities were then related to soil properties and rice productivity.

**Results:**

The rhizospheric bacterial communities were characterized by the phyla Proteobacteria, Acidobacteria, Chloroflexi, and Verrucomicrobia during all stages of rice growth. Rice production differed by approximately 30% between high- and low-yield sites that had uniform fertilization regimes and climatic conditions, suggesting the key role of microbial communities. Mantel tests showed a strong correlation between soil conditions and rhizospheric bacterial communities, and microorganisms had different effects on crop yield. Among the four growing periods, the rhizospheric bacterial communities present during the heading stage showed a more significant correlation (*p* <  0.05) with crop yield, suggesting their potential in regulating crop production. The biological properties (i.e., microbes) reflected the situation of agricultural land better than the physicochemical characterics (i.e., nutrient elements), which provides theoretical support for agronomic production. Molecular ecological network (MEN) analysis suggested that differences in productivity were caused by the interaction between the soil characteristics and the bacterial communities.

**Conclusions:**

During the heading stage of rice cropping, the rhizospheric microbial community is vital for the resulting rice yield. According to network analysis, the cooperative relationship (i.e., positive interaction) between between microbes may contribute significantly to yield, and the biological properties (i.e., microbes) better reflected the real conditions of agricultural land than did the physicochemical characteristics (i.e., nutrient elements).

**Electronic supplementary material:**

The online version of this article (10.1186/s12866-018-1174-z) contains supplementary material, which is available to authorized users.

## Background

Recent studies have suggested that modern agriculture will face substantial challenges over the coming decades [1], and the market demand for agricultural products will increase by at least 70% to 2050 [2]. Over the last few decades, improper agricultural production methods, e.g. the improper use of chemical fertilizers and pesticides [3], have triggered a series of environmental problems [4, 5]. Numerous studies have addressed sustainability issues, and one recommended approach is exploiting the soil microorganisms [2] to sustainably meet agricultural demands [6]. Soil microorganisms play important roles in agriculture, particularly in the nutrient supply and in the biocontrol of plant disease [7, 8]. Rhizospheric microorganisms exist within a narrow zone around the root of a plant and are found at densities of approximately 10^11^ cells per gram [9];additionally, these microorganisms, are considered as the plant’s second genome [10]. The rhizospheric microbial community structure is mainly mediated by root exudate (e.g., sugars, amino acids, siderophores, and enzymes) [11]. Therefore, the interaction between plant roots and rhizospheric microorganisms potentially influences ecosystem functioning by promoting the circulation of materials [12].

Complex biochemical processes occur between rhizospheric microorganisms and plants, and microorganisms enhance soil fertility [13–15], maintain below-ground ecological structure and are associated with plant health (e.g., diseases, pathogens and weed suppression) [16]. Microbial diversity is also an indicator of soil microorganisms in a region [17], which provide a vast amount of ecological information in terms of the soil. Although the relationships between the soil microbial diversity and the functioning and sustainability of agricultural ecosystems have not been fully elucidated [18, 19], it is accepted that microbial diversity plays an important role in agricultural production [20, 21]. Microbial diversity includes the range of microorganisms and their relative abundance in natural habitats [22]. In addition to microbial diversity, microbial community structure also responds to the basis of agroecosystem services in agricultural production [23]. Soil microbial communities drive globally important processes [24], including elemental cycles and energy flows. These microbial communities are involved in various processes that serve essential functions in agricultural production [25] by promoting crop absorption of nutrients and inhibiting harmful pathogens [7, 8, 26–28]. For example, soil microbes can promote plant growth through the degradation of manure fertilizers and form humus nutrients, which are then easily absorbed by plants. Other microorganisms may regulate soil pH, generating favourable conditions that permit functional microorganisms to work at full capacity and promote production [29]. This kind of microorganism often plays a crucial role in the agricultural ecosystem. Explaining the direct effects of soil microbial communities on crop-growth and yield is challenging because the ecosystem functions provides by most soil microorganisms are not well clear [30, 31]. Numerous studies have shown dramatically related results that indicate soil community structure is characterized by Proteobacteria, Acidobacteria, Actinobacteria, Bacteroidetes, Firmicutes, etc. Nevertheless, further consideration of the interactions between the microbial communities and the external environment has identified biological and abiotic factors related to crop yield. Some studies have associated the soil microbial properties with the transformation of edaphic nutrients, which have an essential role in plant growth that helps obtain high yields [10, 32]. However, the innate mechanism of how microorganisms specifically affect crop productivity is poorly understood, and our study is devoted to statistically explaining the links between them.

In the process of production, we found a gap (more than 30%) in crop production between different super rice cultivation types with similar, fertilisation regimes and latitudinal and longitudinal positions. High-throughput sequencing methods served to collect rhizospheric microbial community information in soils. The relationship between soil physicochemical attributes and rhizospheric microorganisms at different stage (from pre-transplanting to the ripening stage) of the super hybrid rice “Y Liang You 900” was investigated to reveal the inborn mechanism of how microorganisms specifically affect crop productivity. We mainly explored the interaction of microbial communities. By comparing the differences between soil microorganisms in high- and low-yield areas during discrete periods, we explored the mechanism of how microorganisms specifically affect agricultural productivity based on soil characteristics. Our study concluded that (1) it is possible improve the average crop yield by controlling rational agricultural management in the heading stage, (2) the positive species interactions within communities may contribute to crop yield, and(3) the biological properties (i.e., microbes) were better than the physicochemical characteristics (i.e., nutrient elements) in terms of reflecting the actual situation of agricultural land.

## Methods

### Study sites and sampling

Soil samples were collected from Xupu (110°31′E, 27°23′N, Ha), Ningxiang (112°16′E, 28°08′N, Ly), Longhui-Zhaojiachong (110°56′E, 27°27′N, Hb) and Longhui-Niuxingzui (110°56′E, 27°29′N, Hc). The sites are paddy fields planted with “Y Liang You 900” super hybrid rice. Sampling was conducted on private land, and the land owner gave permission for our sampling activity. For all sites, the fertilization regime was the same and rice was harvested in October, 2014. The rice yield was calculated according to Chen et al. [33]. Rhizospheric soil samples were collected at four rice development stages: pre-transplanting stage (0 weeks), tillering stage (6 weeks), heading stage (14 weeks), and ripening stage (20 weeks). When sampling, three biological replicates were sampled from each site, resulting in a total of 48 samples (4 stages × 4 sites × 3 replicates = 48 samples, also see in Additional file 1: Table S2). Rhizosphere soils were sampled according to Smalla et al. [34]. After sampling, soil samples were separated into two parts, one part was air dried and stored at 4 °C until physiochemical analysis and the other part was frozen in liquid nitrogen and stored at − 80 °C until molecular analysis.

The yields of super hybrid rice “Y Liang You 900” from four sampling sites are presented in Additional file 1: Table S1. Based on the rice yield, the sites were referred to as ‘low-yield’ sites (Ly, 1.074 kg/m^2^) and ‘high-yield’ sites (Ha, 1.543 kg/m^2^; Hb, 1.517 kg/m^2^; and Hc, 1.589 kg/m^2^). The yield of ‘high-yield’ sites was approximately 30% higher than the yield of the ‘low-yield’ site.

### Soil properties

Soil pH was measured using a pH metre and by dissolving 5 g of each soil sample in 25 mL of distilled water. The nitrogen contents, including total nitrogen (TN) and available nitrogen (AN), were determined by the Kjeldahl procedure [35]. Phosphorus (P) was determined photo metrically as orthophosphate with using a vanado-molybdate method [36], and potassium (K) was determined using inductively coupled plasma-atomic emission spectroscopy (ICP-AES) [37]. Additionally, soil organic matter (SOM) was analysed using the potassium dichromate method by titration with ammonium ferrous sulphate (i.e., Mohr’s salt solution) [38].

### DNA extraction, PCR amplification and MiSeq sequencing

The soil samples were homogenized in liquid nitrogen and mixed completely, and 1 g of each soil sample was used for DNA extraction. Soil microbial genomic DNA was extracted using a soil microbial DNA extraction kit (MOBIO, San Diego, USA). The hyper-variable region (V4) of prokaryotic 16S rRNA [39] was amplified using the primer pair 515F (5′-GTGCCAGCMGCCGCGGTAA-3′) and 806R (5′-GGACTACHVGGTWTCTAAT-3′) [31]. PCR products were recovered and the quality and quantity of recovered PCR products was determined using a Nano-drop spectrophotometer (Thermo Fisher Scientific, Waltham, MA, USA). Purified PCR products were subjected to the MiSeq platform (Illumina, San Diego, CA) with a 500-cycle kit (2 × 250 bp paired-ends) for sequencing. Data processing was conducted according to Tao et al. [40] and Yin et al. [41]. Specifically, reads were assigned to different samples according to the barcode sequence and primers were removed. The left and right reads were then merged with a minimum of 10 bp overlap and less than 5% mismatches using Flash [42]. Ambiguous bases (N) were removed from the merged sequences and chimers were removed by Uchim [43]. Finally, high-quality sequences were clustered using UCLUST [44] at 97% similarity level [45], and the OTU table was constructed after removing the false positive sequences (singletons). Taxa assignment was carried out by blasting the sequences to the RDP database [46] with 50% confidence. The rarefaction curve is shown in Additional file 1: Figure S1. To reduce the variations caused by different sequencing depth, sequencing depths was rarefied to16, 000 for all samples and the rarefied OTU table was used for all downstream analyses. All sequences were submitted to the NCBI database under the accession number SRP083104.

### Statistical analysis

Soil microbial diversity indices, including the Shannon Wiener, Inverse Simpson, and Pielou evenness indices, were calculated using the R version 3.3.2 (https://www.r-project.org/) platform with the package vegan 2.4.2. [47]. Detrended correspondence analysis (DCA) for detecting the variation in microbial community composition among sites/stages was also performed using the vegan package. Permutational multivariate analysis of variance (Adonis) was used to test the effects of soil variables on crop yield. Standard and Mantel tests were carried out to identify the correlations between environmental factors and soil bacterial communities (based on Euclidean distance). The relationship between soil properties and microbial microorganisms in the heading stage was further analysed by the PLSPM model (partial least-squares path) using the plspm package [48]. Additional statistical analysis including one-way ANOVA and Pearson correlation analysis was also completed using R version 3.3.2, and a *p* value less than 0.05 was considered significant. A molecular ecological network (MEN) using an RMT-based network approach was built on the IEG website (http://ieg4.rccc.ou.edu/) to investigate the interaction between microbes [49–51] and the networks; finally, the result were visualized using Cytoscape 3.4.0.

## Results

### Soil properties

**Fig. 1:**
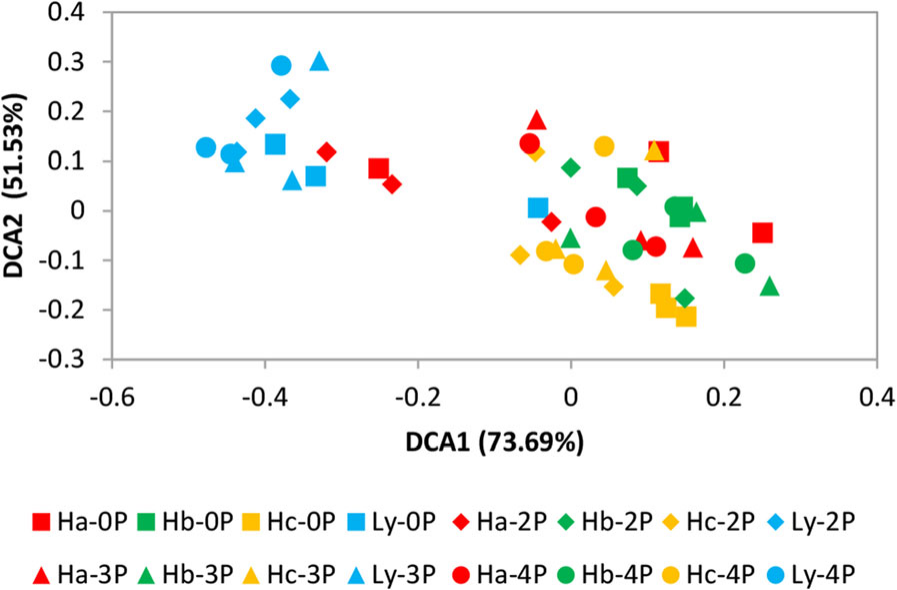
Detrended correspondence analysis (DCA) of soil environmental factors with all samples from pre-transplanting stage to ripening stage

The pH varied from 4 to 7 (Additional file 1: Table S3), and the lowest pH was at the Ly site. The Ly site had a lower concentration of soil nutrients (N, P, and K) than the other sites; particularly, the SOM (soil organic matter) showed the largest difference between Ly and the high-yield sites. Permutational multivariate analysis of variance (Adonis) was conducted using three methods (Bray, Euclid and Horn), and analyses indicated that the rice yield was closely associated with the soil physiochemical properties (Additional file 1: Table S4). The DCA based on soil environmental factors (Fig. 1) showed the low-yield site had very different soil properties compared to the high-yield sites. Pearson correlation analysis showed various correlations between the rice yield and the soil properties (Additional file 1: Table S5). Among all soil properties, the available nitrogen (AN) and available potassium (AK) were significantly (Pearson correlation > 0.650; *p* <  0.05) and positively associated with rice yield. In addition, TN, TP, and SOM were also significantly correlated (*p* <  0.05) with crop yield in all stages of development. Furthermore, pH was substantially correlated with yield during the tillering stage.

### Bacterial diversity

**Fig. 2:**
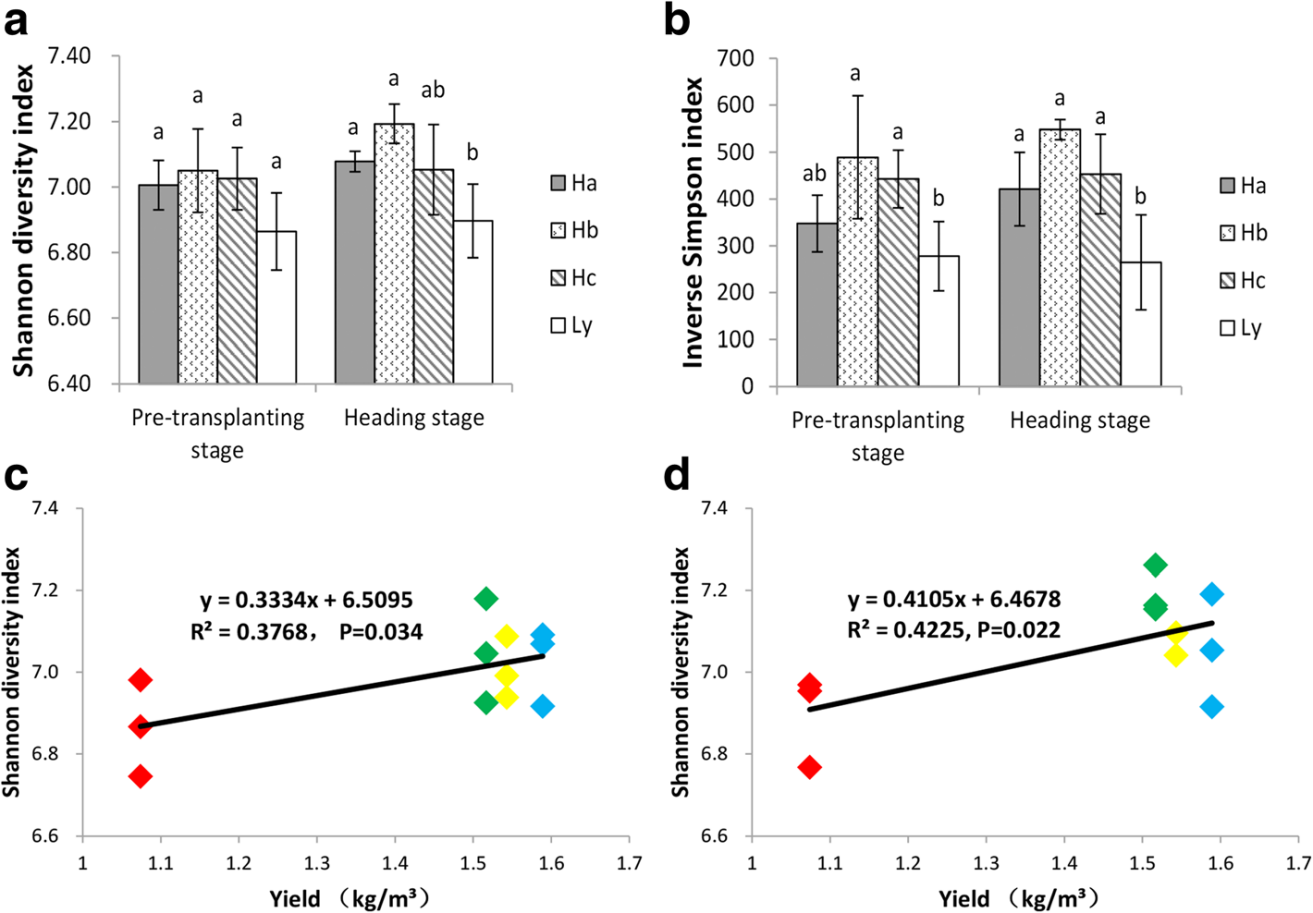
Dcomparing the differences in microbial diversity indices was carried out by one-way analysis of variance (ANOVA) before the transplanting and heading stages. Error bars are based on the standard error and different lowercase letters indicate significant differences at the level of 0.05 as indicated by the ANOVA results. (**a**): Shannon Wiener index; (**b**): Inverse Simpson index; (**c**)Linear regression analysis of the relationship between crop yield and Shannon Wiener diversity index during pre-transplanting stage; and (**d**) Linear regression analysis of the relationship between crop yield and Shannon Wiener diversity index at the heading stage

The microbial community diversity indices, including Shannon and Simpson diversity and Pielou evenness were significantly higher in the high-yield sites than in the low-yield site, and generally, the diversity indices were the highest during the tillering stage. The diversity differed slightly between the pre-transplant and heading stages in the Ha and Hc sites; however, the bacterial diversity at the Ly site was similar during all stages of crop development. In addition, two-way ANOVA indicated that both stage and site had significant effects on microbial community diversity indices (Table 1). Pearson correlation showed the microbial community diversity during the pre-transplanting and heading stages was positively correlated (*p* <  0.05) with rice yield (Fig. 2). Adonis (permutational multivariate analysis of variance) also showed the microbial community during the pre-transplanting stage and the heading stage had a significant effect on crop yield (Additional file 1: Table S6), whereas the tillering and ripening stages did not show significant effects.

**Table 1:** Rhizospheric microbial community diversity at different developmental stages at four sites

	Shannon diversity	Simpson	Pielou evenness
Ha_0p	7.01 ± 0.08abcde	0.9971 ± 0.0005ab	347.61 ± 60.66abcd
Ha_2p	7.28 ± 0.11a	0.9981 ± 0.0004a	553.05 ± 116.91a
Ha_3p	7.08 ± 0.03abcd	0.9976 ± 0.0005ab	421.17 ± 78.66abcd
Ha_4p	6.77 ± 0.05e	0.9948 ± 0.0026b	223.77 ± 95.43d
Hb_0p	7.05 ± 0.13abcde	0.9978 ± 0.0006ab	488.8 ± 131.09abc
Hb_2p	7.28 ± 0.06a	0.9983 ± 0.0002a	583.58 ± 62.07a
Hb_3p	7.19 ± 0.06ab	0.9982 ± 0.0001a	548.53 ± 21.6ab
Hb_4p	7.17 ± 0.01abc	0.9982 ± 0.0001a	551.81 ± 16.64ab
Hc_0p	7.03 ± 0.1abcde	0.9977 ± 0.0003ab	442.72 ± 61.61abcd
Hc_2p	7.07 ± 0.17abcde	0.9975 ± 0.0009ab	427.48 ± 139.1abcd
Hc_3p	7.05 ± 0.14abcde	0.9977 ± 0.0005ab	453.29 ± 85.08abcd
Hc_4p	6.87 ± 0.13cde	0.9969 ± 0.0005ab	328.54 ± 58.07bcd
Ly_0p	6.86 ± 0.12de	0.9962 ± 0.0012ab	277.73 ± 73.87bcd
Ly_2p	6.94 ± 0.1bcde	0.9971 ± 0.0007ab	363.47 ± 92.08abcd
Ly_3p	6.9 ± 0.11bcde	0.9957 ± 0.0021ab	264.65 ± 101.48bcd
Ly_4p	6.84 ± 0.04de	0.996 ± 0.001ab	257.85 ± 56.84 cd
Two-way ANOVA	Shannon	Simpson	Pielou evenness
Site effect	< 0.001	0.001	< 0.001
Stage effect	< 0.001	0.034	< 0.001
Cross effect	0.026	0.182	0.082

0p: pre-transplanting stage; 2p: tillering stage; 3p: heading stage; and 4p: ripening stage. The results are shown as the means and S.D. of three biological replicates. Values that do not share letters are different at the *p* < 0.05 level following Tukey’s t-test. Site, stage and cross effects were accessed by two-way ANOVA

### Network analysis on relationships between key microbial communities

**Fig. 3:**
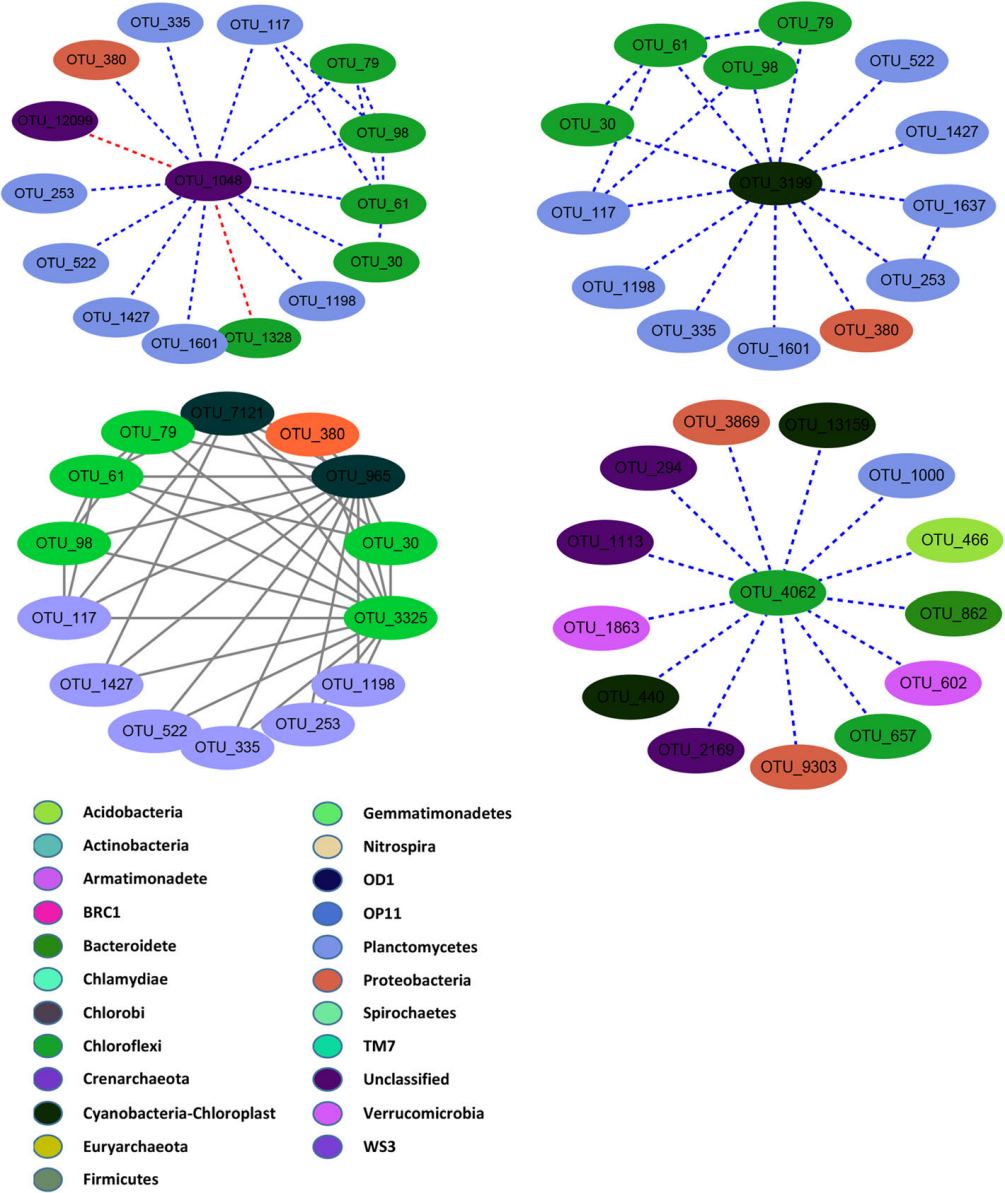
Network constructed by thethe highest level (highly degree) of bacterial communities at site Hc. Each node signifies an OTU that could corresponds to a microbial population. Colours of the nodes indicate different major phyla. Blue and red lines represent positive and negative path coefficients, respectively

**Fig. 4:**
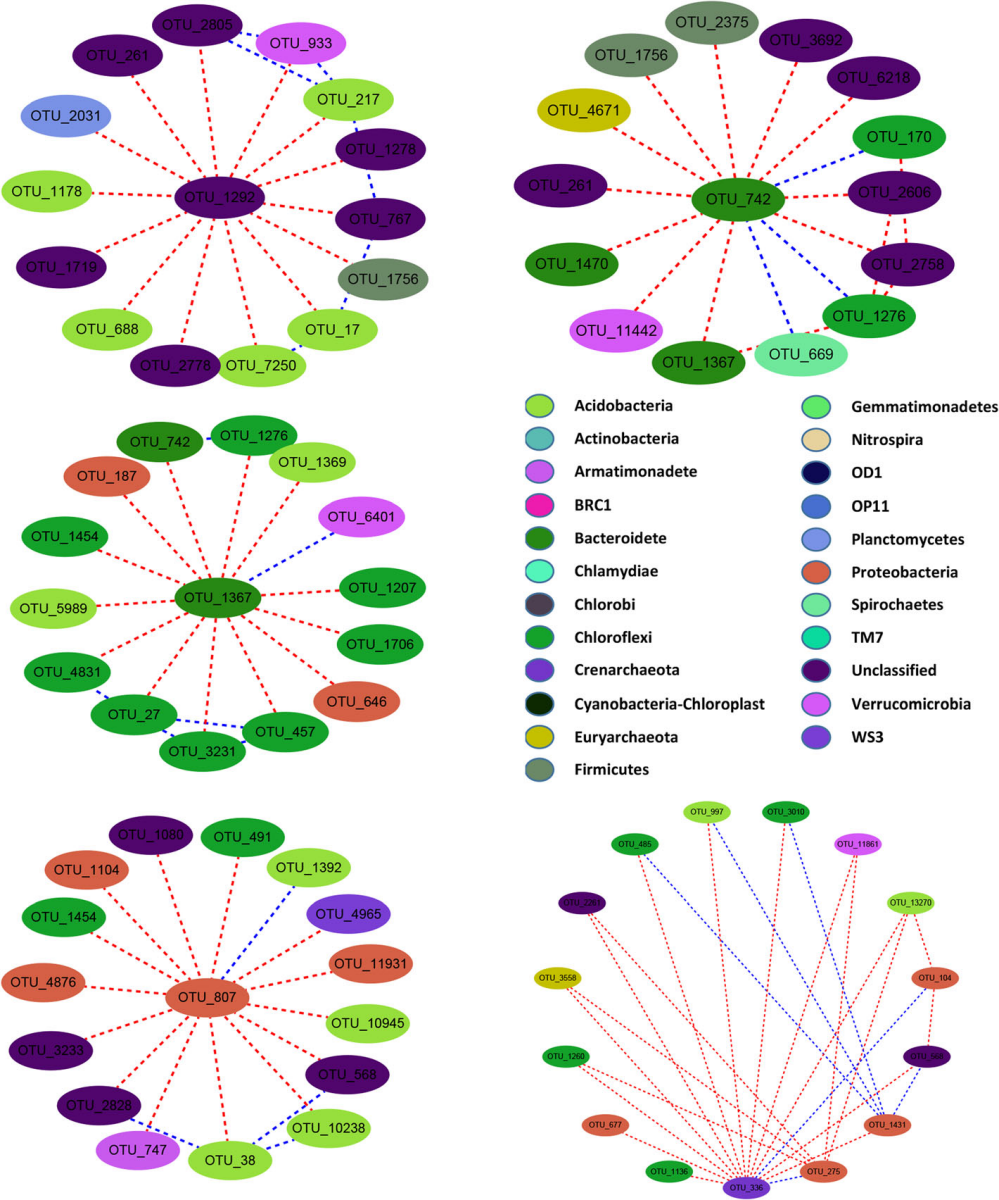
Network constructed by thethe highest level (highly degree) of bacterial communities in site Ly. Each node signifies an OTU that corresponds to a microbial population. Colours of the nodes indicate different major phyla. Blue and red lines represent positive and negative path coefficients, repectively

To explore the interactions between rhizospheric microbes, RMT-based molecular ecological networks (MEN) were constructed and analysed. All sites had similar RMT thresholds (0.88~ 0.90). However, the number of nodes and links was lower in the Ly site than in the other sites (Figs. 3 and 4, Additional file 1: Figures S2 and S3). There were more positive interactions between OTUs in the high-yield site (Ha, Hb and Hc) network than that in the low-yield site (Ly) network. Nodes with high degrees in the Ly networks were classified into *Bacteroides* (OTU_1367 and OTU_742) and *Proteobacteria* (OTU_807 and OTU_275), while in high-yield site networks, nodes with high degree were classified into *Acidobacteria*, *Actinobacteria* and *Planctomycete*. In addition, site Hb had the most complicated network (Additional file 1: Figure S3) with the most links and the maximal degree, presenting more intricate topological properties than the other sites.

### Microbial community composition and structure

**Fig. 5:**
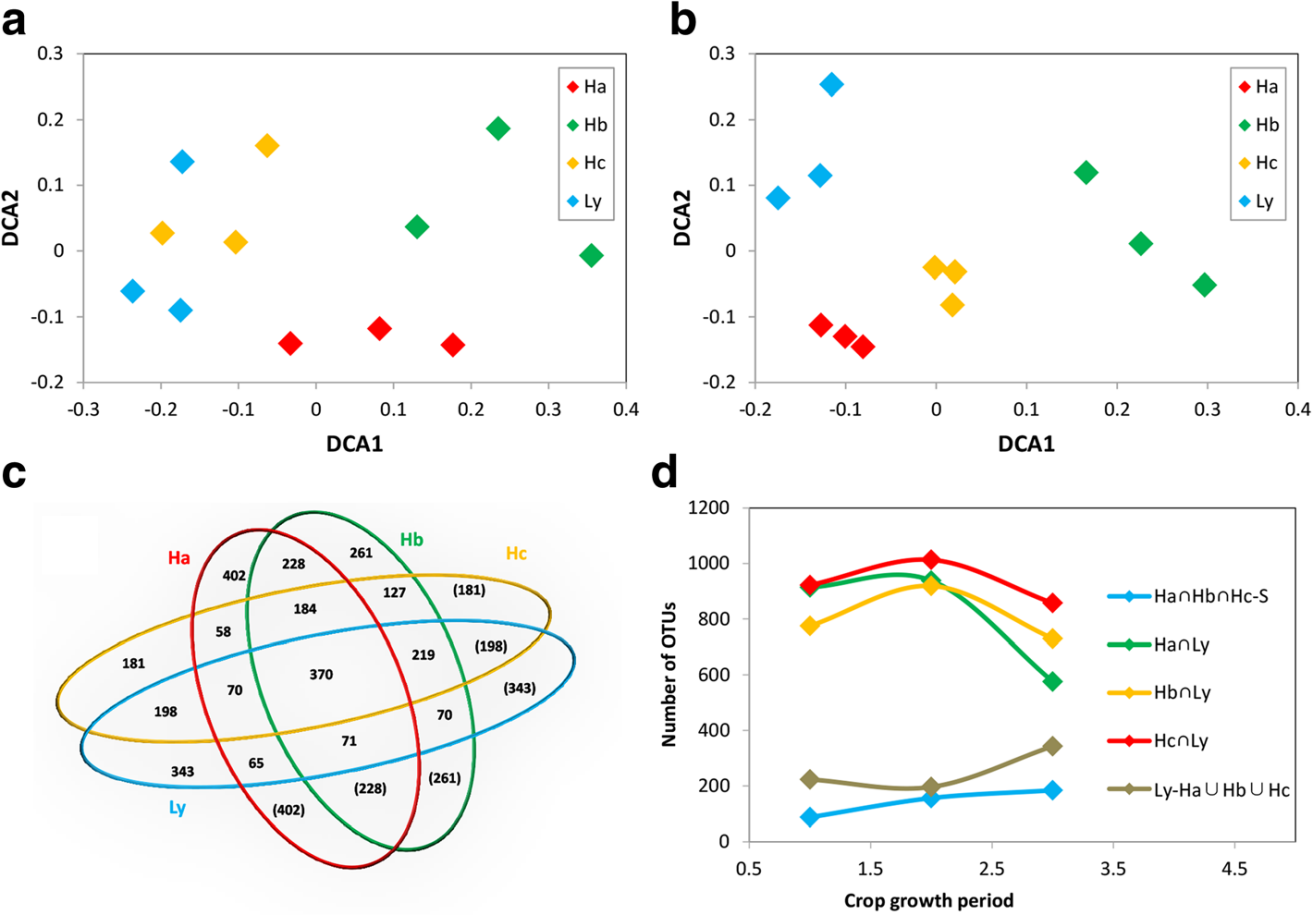
Analsis of compositions and structures of bacterial communities from four groups. (**a**) Detrended correspondence analysis (DCA) of 16S rRNA gene sequencing data at the genus level during the tillering stage; (**b**) Detrended correspondence analysis (DCA) of 16S rRNA gene sequencing data at the genus level during the heading stage; (**c**) Venn diagrams were calculated by R with the package gplots and based on OTU level during the heading stage. Figures in pictures represent the taxa number of OTUs with common ownership at different sites; (**d**) Variation trends in OTUs under different classifications from pre-transplanting to heading stages. ∩: Intersection of mathematical symbol; ∪:Union mathematical symbol; and S: Ha∩Hb∩Hc∩Ly (intersection of four sites)

All sequences were clustered into 14,332 operational taxonomic units (OTUs) and were assigned into 469 genera. The permutational multivariate analysis of variance (Adonis) from three methods (Bray, Euclid and Horn) at the OTUs (Additional file 1: Table S7) and the genus level (Additional file 1: Table S8) showed that the microbial community structure during the heading stage was closely associated with the yield. A venn diagram (Fig. 5c and Additional file 1: Figure S4) showed there were 87 OTUs shared by sites Ha, Hb, and Hc during the pre-transplanting stage, but this was not found at the Ly site. During the crop-growth period, 184 OTUs were shared by high-yield sites. Whereas the number of shared OTU taxa in the four sites reduced from 600 during the pre-transplanting stage to 370 during the crop-growth period. The number of unique OTUs in site Ly and the other sites increased when the stage changed from the pre-transplanting stage to the heading stage (Fig. 5d). An increase in unique OTUs in site Ly indicated that the difference between high- and low-yield sites increased. Detrended correspondence analysis (DCA) showed that the microbial community structure differed more substantially during three of the crop-growth stages than in the pre-transplanting stage (Fig. 5a and b; Additional file 1: Figure S4c and d). Furthermore, the community structure differences between the low- and high-yield sites were most obvious during the heading stage; therefore, we focused on the rhizospheric microbial community during the heading stage (Additional file 1: Table S9). Proteobacteria, Acidobacteria, and Chloroflexi were the most abundant phyla found in the rhizospheric microbial community during the heading stage (Additional file 1: Table S9a), and each accounted for more than 10% of the community. At the class level, Deltaproteobacteria was the most abundant class in all samples, and was followed by Anaerolineae*, Betaproteobacteria,* Alphaproteobacteria. (Additional file 1: Table S9b).

### Relationship between soil characteristics, microorganisms and crop yield

Mantel tests were carried out to determine the correlations between the soil physical and chemical properties and the soil microorganisms [52] (Table 2). The results indicated that soil conditions had a significant influence on the bacterial community structure (*p* <  0.05) during the pre-transplanting and heading stages. During the heading stage, there was a significantly negative correlation (Pearson correlation = − 0.647; *p* <  0.05, Additional file 1: Table S10) between the yield and Crenarchaeota (Additional file 1: Table S10). In addition, soil environmental factors, such as AK, had a significant negative correlation (Pearson correlation = − 0.666, *p* <  0.05; Additional file 1: Table S10) with Crenarchaeota, but a significant positive correlation with crop yield. Crop yield and AN were also significantly positively correlated (Pearson correlation = 0.668; *p* < 0.05; Additional file 1: Table S10). In addition, Proteobacteria and Bacteroidetes showed a significant positive correlation (Pearson correlation = 0.743–0.752; *p* < 0.01; Additional file 1: Table S10) with pH, whereas Chloroflexi exhibited a significant negative correlation with pH (Pearson correlation = − 0.621; *p* < 0.05, Additional file 1: Table S10). Overall, pH showed significant effects on constraining the bacterial community.

**Table 2:** Pearson correlation between microbial community diversity and rice yield

Pearson correlation	Yield			
	Before transplanting	Tillering stage	Heading stage	Ripening stage
Shannon.Wiener	0.614*	0.613*	0.650*	0.197
Simpson	0.579*	0.485	0.720**	0.286
Pielou	0.658*	0.477	0.594*	0.182

The stars indicate the significance level: *: *P* < 0.05, **: *P* < 0.01

Further analyses showed 17 phyla had significant effects on soil fertility and crop production. Some genera had significant impact on soil fertility as shown in Additional file 1: Table S11. The genera *Geobacter, Syntrophorhabdus, Phaselicystis, Thiobacter*, and *Chondromyces* (in addition to *Cystobacter*) of the phylum Proteobacteria, had negative impacts on crop yield. However, the abundance of the Proteobacteria was positively correlated with soil fertility. This also stressed that the structure and function of the bacterial community was diverse and complex at the phylum level. *Armatimonadetes*-gp2 and *Armatimonadetes*-gp5 in *Armatimonadetes* showed contradictory correlation with soil fertility and crop production. *Gp17, Gp6, Gp24,* and *Gp25*, which belong to *Acidobacteria*, were significantly corelated with production. *Spartobacteria incertae sedis* in *Verrucomicrobia*, and *OD1-sedis* in *OD1* showed diverse effects on crop yield. Therefore, the overall effect could be directly observed at the phylum level, as well as on a more precise level when considering the individual effects of each bacterial community at the genus level.

**Fig. 6:**
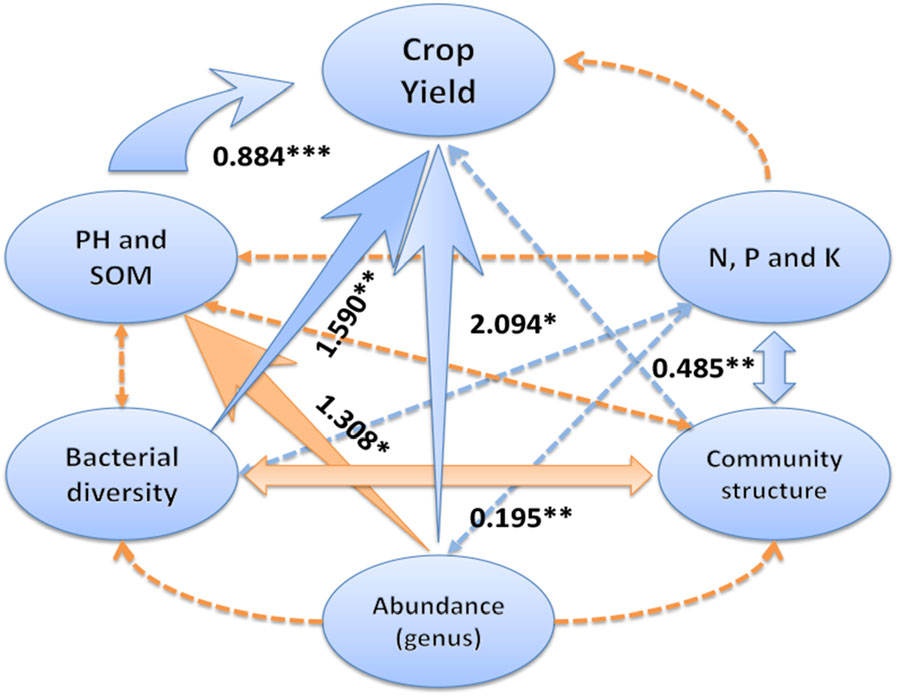
Partial least squares path modelling (PLSPM) of the association between the yield of super rice and soil biological and abiotic factors during the heading stage. Goodness-of-fit of the model is 0.702. Blue and orange arrows represent positive and negative path coefficients, respectively. *p < 0.05, **p < 0.01, **p < 0.01

The partial least squares path model (PLSPM, Fig. 6) showed an association between the yield and soil biological and abiotic factors, in general, at the heading stage. The bacterial diversity and the major bacterial communities were substantially related to the yield of super rice, but the structure of the bacterial communities was insignificant. The main soil factors affecting the yield of super rice were pH and SOM, which were also closely associated with the structure of soil bacterial communities. The goodness of fit to the model was 0.702 (> 0.350), which validates the result and provides a reference value for our study.

## Discussion

### Relationship between rhizospheric microbial community and rice productivity

Crop productivity was mainly affected by the flow of energy and material in the soil ecosystem which is driven by soil organisms. Microorganisms located in the plant’s rhizosphere play pivotal roles in the soil geochemical cycle [53]. In the present study, the microbial community responded instantly to ecological changes during the pre-transplanting and heading stages. Generally, the bacterial diversity was higher in the high-yield sites than in the low-yield site, which suggested that high bacterial diversity potentially increases the yield of super rice. This is because high bacterial diversity maintained a relatively stable ecosystem in the rhizosphere, which allowed effective nutrient cycling [54]. The difference in the microbial communities between the high-yield sites and the low-yield site may be a result of different nutrient levels in the soils. This was further supported by the Mantel test, which showed that the soil physiochemical properties had significant effects on the soil microbial communities. Resident plants shaped and restructured rhizospheric microbial communities via root exudates [55], which provides nutrients for microbial communities and regulated microbial diversity [56, 57]. The ecosystem properties such as robustness and trophic interactions [58] were more stable in response to environmental fluctuations as diversity increased, for the richness of taxonomic diversity leads to the extension of niches and the utilization of resources. It is urgent to improve the stability and sustainability of farmland ecosystems [59], and meagre microbial diversity makes it difficult to resist the interference of detrimental factors. Additionally, a previous study by Laurent Philippot (2013) showed a loss in microbial diversity could affect nutrient cycling in soil [54], and microbial communities were closely related to material cycling.

### Rhizospheric microbial community was crucial for rice production in the heading stage

The heading stage is a critical period for crop production; during this time, crops exhibit the most rapid growth and development of their entire life. It is a critical period for determining the amount of grains. During this stage, crops respond to external conditions [60]. Abundant nutrients, water, temperature, illumination, and other external conditions are required during this period, suggesting that this is an excellent time for farmers to increase production by means of topdressing. Soil fertility, a fundamental factor for plant growth, is mainly dependent on microbial transformations. Thus it is not surprising that the microbial community was most closely related to rice production during the heading stage. The association between the super rice with soil characteristics and the microbial microorganisms during the heading stage was further analysed using the PLSPM (partial least-squares path, Fig. 6). Bacterial communities such as *Blastopirellula* in the Planctomycete, are good indicators of soil fertility because the microorganisms respond to topdressing more rapidly than do the soil physicochemical characteristics [61], and microorganisms represent the true situation of the farmland; this is of great significance when establishing a stable and high-yield agricultural system. In the present study, some bacterial communities such as Chloroflexi, which do not produce oxygen during photosynthesis and have decreased nitrogen-fixation abilities, are abundant in soils; therefore, these bacteria are negatively correlated with the yield of super rice [62]. In contrast to detrimental bacteria, some bacterial communities, such as members of Acidobacteria, can degrade cellulose and adjust soil pH effectively [63, 64]; therefore, they are closely related to rice yield. However, the specific features of some *Acidobacteria*, e.g., *GP6* and *GP17*, remain unclear.

### Effects of microbial species interactions on rice productivity

Distinct sites had uniform fertilization regimes and similar climatic conditions, so the main difference in productivity was due to the rhizospheric microbial communities. In agricultural production, rhizospheric microorganisms are always associated with a considerable yield within a stable farmland ecosystem [65]. According to the molecular ecology networks, rhizospheric bacterial communities that played pivotal roles in the site Ly (Fig. 4), such as *Bacteroidetes* and *Proteobacteria*, are always classified as ‘copiotrophs’ (R-strategists), having a high growth rate in nutrient-rich conditions [66]. Owing to efficient metabolism in decomposing organic matter, the R-strategists should be chosen as the centre of communication among microbial communities, especially in artificially controlled nutrient-rich conditions. A farmland is a relatively complete ecosystem that keeps a dynamic balance between the input (contrived fertilizers) and output (plant growth) energetic processes; thus, due to rich resources leading to niche diversification and a reduction in the intensity of competition between co-occurring communities [57], the excellent decomposers and communicators with powerful metabolic rates, i.e., R-strategists, should present a positive role in the node of energy flows and material cycling. After all, co-occurrences may indicate a benign or mutually beneficial relationship. Conversely, the results showed that many R-strategists were negatively linked with their nearest neighbour in site Ly due to competition for resources, which implies a decline in microenvironment vitality. One possible explanation is that mutual exclusivity between bacterial communities caused by competition exists widely in the low-yield area, especially within similar niches. In addition, high-yield sites differed considerably from site Ly in terms of most members of Actinobacteria, Acidobacteria, and Planctomycetes. We infer that member of Acidobacteria and Actinobacteria had stronger adaptability and resilience, which enable their survival under stressful conditions [66], and regulated the surrounding environmental attributes through feedback mechanisms. The microbial community links showed more specific ecological significance than the physicochemical characteristics of farmland throughout the entire period of rice growth.

## Conclusion

The results of the present study demonstrated that the gaps in crop yield are strongly associated with variations in the soil microbial communities during the heading stage, which were due to the effects of soil characteristic before transplanting; thus, in addition to adjusting the microhabitat before transplanting, there is the potential to improve the average crop yield by controlling rational agricultural management during the heading stage. In addition, the positive species interactions within communities may contribute to yield, as seen through network analysis, and the biological properties (i.e., microbes) better reflect real-world farmland situations than do physicochemical characteristics (i.e., nutrient elements). However, we have essentially focused on the relationships among soil characteristics and the variations and interactions of microbial communities. Hypotheses can be formed based on correlation analyses and need to be further tested to determine whether they are applicable to other land types.

## Additional file

Additional file 1: **Figure S1.** Rarefaction curve of 16S rRNA gene sequencing at four sites (sites Ha, Hb, Hc and Ly). **Figure S2.** Network constructed by the highest level (highly degree) of bacterial communities at site Ha. **Figure S3.** Network constructed by the highest level (highly degree) of bacterial communities at site Hb. **Figure S4.** Analysis of compositions and structures of bacterial communities from four groups.**Table S1.** Average yield of super hybrid rice “Y Liang You 900” at four sampling sites. **Table S2.** Name and batch of detected samples. **Table S3.** Mean value ± standard deviation (*n* = 3) for chemical properties of soils during the four periods . **Table S4.** Permutational multivariate analysis of variance (Adonis) shows the effects of soil properties at the various stages of crop transplanting on the yield of super rice using three methods. **Table S5.** Pearson positive correlations between crop yield and several physical and chemical soil properties during the four stages of development. **Table S6.** Permutational multivariate analysis of variance (Adonis) shows the effect of bacterial diversity on crop yield at four stages of development. **Table S7.** Permutational multivariate analysis of variance (Adonis) shows the effect of bacterial community structure at the OUT level on crop yield at four stages of development. **Table S8.** Permutational multivariate analysis of variance (Adonis) shows the effect of bacterial community structure at the genus level on crop yield at four stages of development. **Table S9.** Relative abundances (%) of the dominant bacterial phyla (a) and classes (b) in soil during heading stage. **Table S10.** Significant Pearson correlations between microbial community and soil environmental factors during the heading stage are shown at thephylum level. **Table S11.** Significant Pearson correlations between bacterial communities and soil environmental factors during the heading stage are shown at the genus level. (PDF 2493 kb)

## Abbreviations

Adonis: Permutational multivariate analysis of variance
AN: available nitrogen
DCA: Detrended correspondence analysis
K: potassium
MEN: Molecular ecological network
P: Phosphorus
PLSPM: Partial least-squares path
SOM: Soil organic matter
TN: Total nitrogen
