# Supplementary material for: Environmental factors shaping the diversity of bacterial communities that promote rice production
Source: BMC Microbiol. 2018 Jun 4;18:51. doi: 10.1186/s12866-018-1174-z (PMC5987589; doi:10.1186/s12866-018-1174-z)
Supplement: Supplementary file 1 — Figure S1. Rarefaction curve of 16S rRNA gene sequencing at four sites (sites Ha, Hb, Hc and Ly). Figure S2. Network constructed by the highest level (highly degree) of bacterial communities at site Ha. Figure S3. Network constructed by the highest level (highly degree) of bacterial communities at site Hb. Figure S4. Analysis of compositions and structures of bacterial communities from four groups.Table S1. Average yield of super hybrid rice “Y Liang You 900” at four sampling sites. Table S2. Name and batch of detected samples. Table S3. Mean value ± standard deviation (n = 3) for chemical properties of soils during the four periods . Table S4. Permutational multivariate analysis of variance (Adonis) shows the effects of soil properties at the various stages of crop transplanting on the yield of super rice using three methods. Table S5. Pearson positive correlations between crop yield and several physical and chemical soil properties during the four stages of development. Table S6. Permutational multivariate analysis of variance (Adonis) shows the effect of bacterial diversity on crop yield at four stages of development. Table S7. Permutational multivariate analysis of variance (Adonis) shows the effect of bacterial community structure at the OUT level on crop yield at four stages of development. Table S8. Permutational multivariate analysis of variance (Adonis) shows the effect of bacterial community structure at the genus level on crop yield at four stages of development. Table S9. Relative abundances (%) of the dominant bacterial phyla (a) and classes (b) in soil during heading stage. Table S10. Significant Pearson correlations between microbial community and soil environmental factors during the heading stage are shown at thephylum level. Table S11. Significant Pearson correlations between bacterial communities and soil environmental factors during the heading stage are shown at the genus level. (PDF 2493 kb) [file 12866_2018_1174_MOESM1_ESM.pdf]

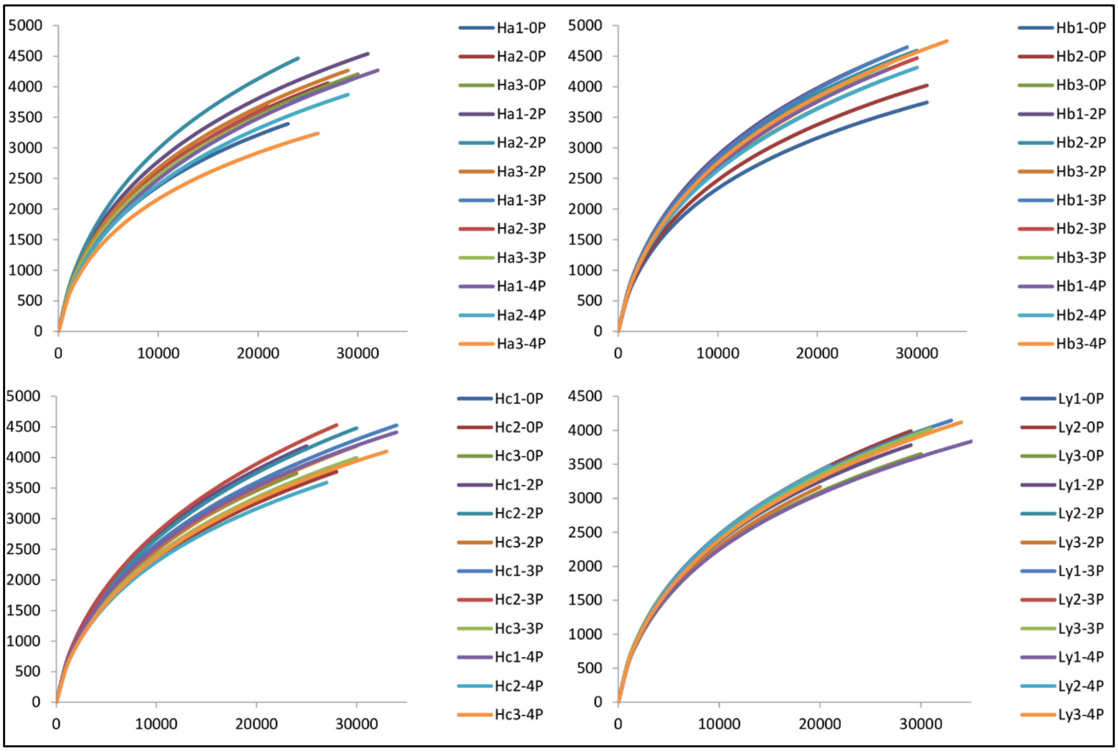

Figure S1 Rarefaction curve of 16S rRNA gene sequencing in four sites (site Ha, Hb, Hc and Ly)

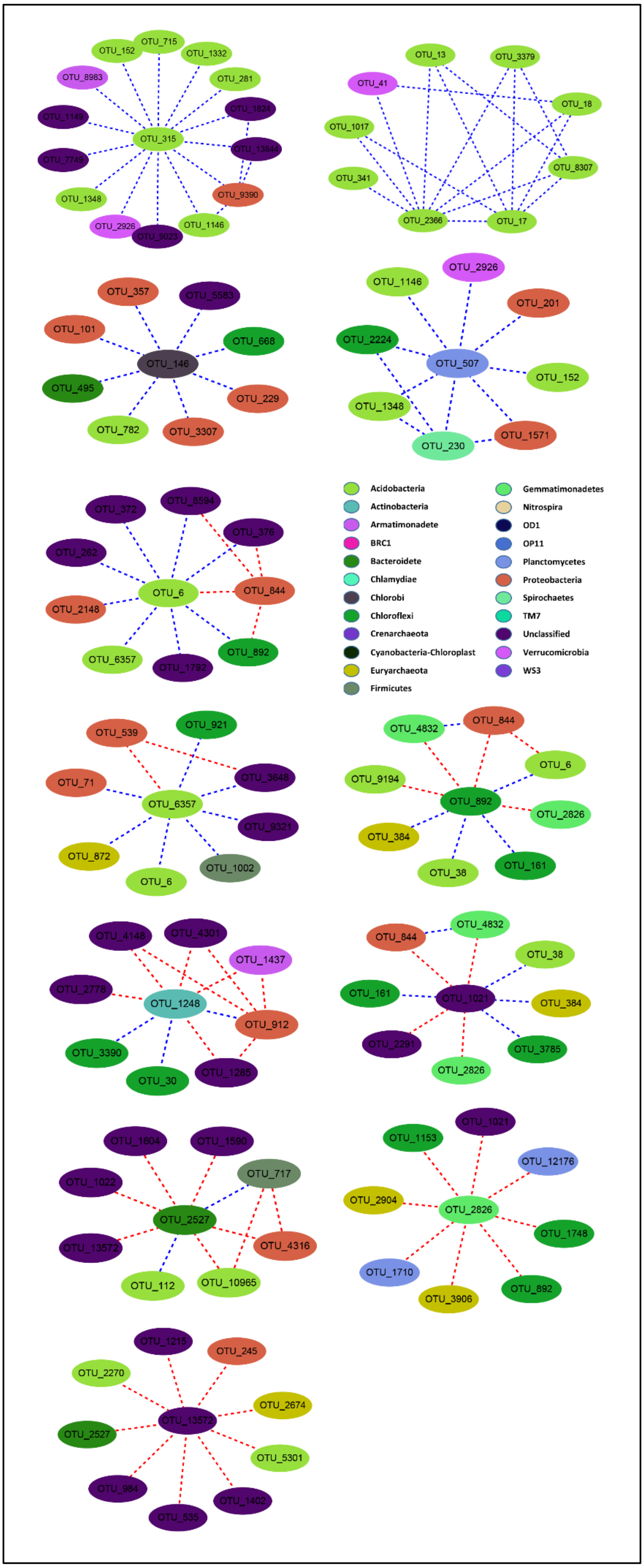

Figure S2 Network constructed by the the highest level (highly degree) of bacterial communities in site Ha. Each node signifies an OTU which could correspond to a microbial population. Colours of the nodes indicate different major phylum. Blue and red lines are respectively on behalf of positive and negative path coefficient.

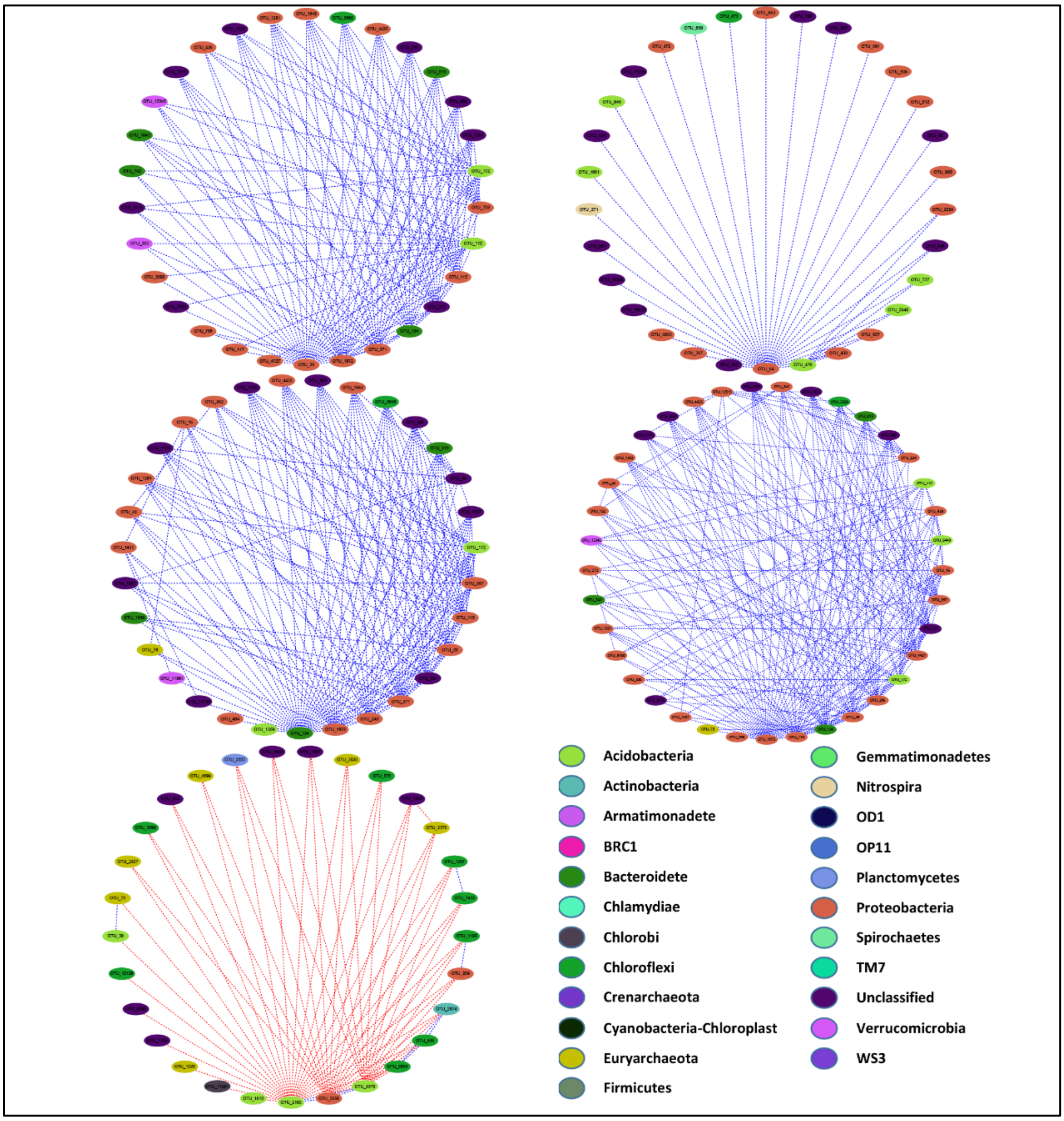

Figure S3 Network constructed by the the highest level (highly degree) of bacterial communities in site Hb. Each node signifies an OTU which could correspond to a microbial population. Colours of the nodes indicate different major phylum. Blue and red lines are respectively on behalf of positive and negative path coefficient.

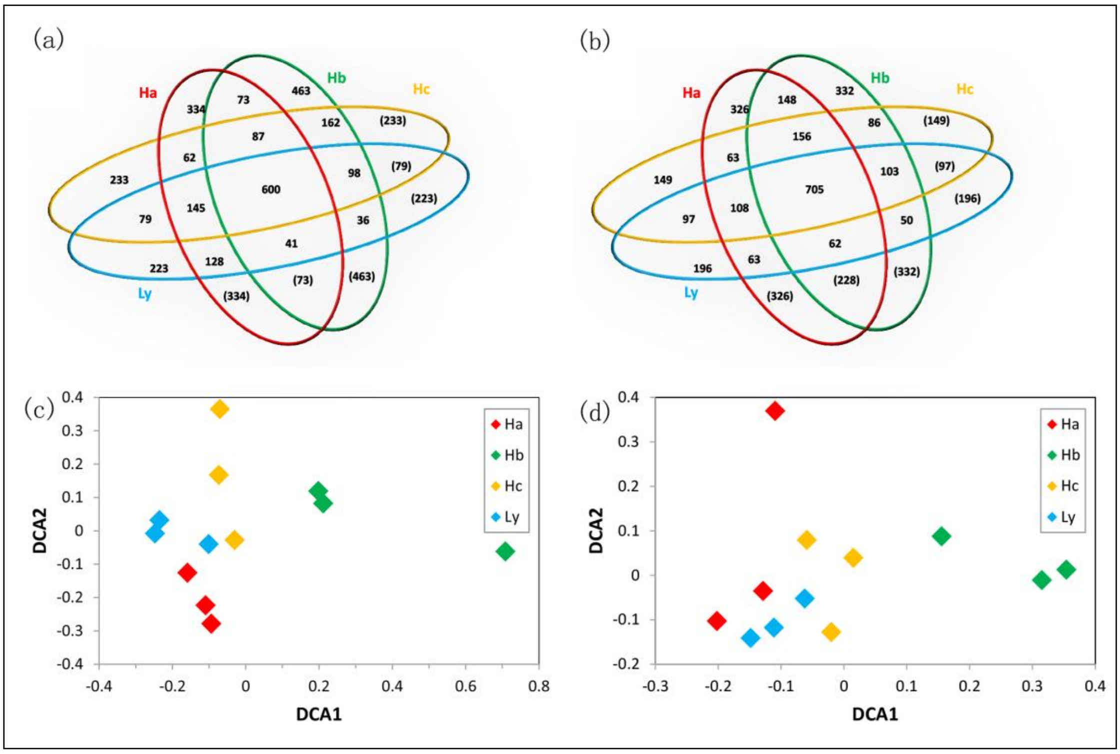

Figure S4 Analsis of compositions and structures of bacterial communities from four groups. (a) Venn diagrams were calculated by R with packages gplots, based on OTUs' level in pre-transplanting stage; (b) Venn diagrams were calculated by R with packages gplots, based on OTUs' level in tillering stage. Figures in pictures represent the taxa number of OTUs of common ownership in different sites; (c) Detrended correspondence analysis (DCA) of 16S rRNA gene sequencing data at genus level in pre-transplanting stage; (b) Detrended correspondence analysis (DCA) of 16S rRNA gene sequencing data at genus in ripening stage;

Table S1 Average yield of super hybrid rice “Y Liang You 900” in four sampling sites.

**Sites** / **Production**	**XuPu** **（Ha）**	**LonghuiZhaojiachong** **（Hb）**	**LonghuiNiuxingzui** **（Hc）**	**NingXiang** **（Ly）**
**Crop Yield** **（kg/m^2）**	1.543±0.018a	1.517±0.017a	1.589±0.045a	1.074±0.058b

Table S2 Name and batch of tested samples. The number of 1, 2 and 3 are on behalf of the same region to collect three duplicate samples, respectively.

**Sampling site**	**0P (Pre-transplanting)**	**2P(Tillering stage)**	**3P (Heading stage)**	**4P (Ripening stage)**
XuPu **(Ha)**	Ha1-0P	Ha1-2P	Ha1-3P	Ha1-4P
	Ha2-0P	Ha2-2P	Ha2-3P	Ha2-4P
	Ha3-0P	Ha3-2P	Ha3-3P	Ha3-4P
LonghuiZhaojiachong **(Hb)**	Hb1-0P	Hb1-2P	Hb1-3P	Hb1-4P
	Hb2-0P	Hb2-2P	Hb2-3P	Hb2-4P
	Hb3-0P	Hb3-2P	Hb3-3P	Hb3-4P
LonghuiNiuxingzui **(Hc)**	Hc1-0P	Hc1-2P	Hc1-3P	Hc1-4P
	Hc2-0P	Hc2-2P	Hc2-3P	Hc2-4P
	Hc3-0P	Hc3-2P	Hc3-3P	Hc3-4P
NingXiang **(Ly)**	Ly1-0P	Ly1-2P	Ly1-3P	Ly1-4P
	Ly2-0P	Ly2-2P	Ly2-3P	Ly2-4P
	Ly3-0P	Ly3-2P	Ly3-3P	Ly3-4P

Table S3 Mean value ± standard deviation (n=3) for chemical properties of soils during the four periods (pre-transplanting, tillering stage and heading stage). Variables with significant ANOVA (α=0.05， n=4) were analyzed by R and the differences among four sites are indicated by lowercase letters. PH: the value of pH when the soil was dissolve in water; AN: available nitrogen; AP: available phosphorus; AK: available potassium; TN: total nitrogen; TP: total nitrogen; TK: total potassium; and SOM: soil organic matter.

**Samples**		**pH**	**AN(mg/kg)**	**AP(mg/kg)**	**AK(mg/kg)**	**TN(g/kg)**	**TP(g/kg)**	**TK(g/kg)**	**SOM(g/kg)**
**Pte-transplanting stage (0P)**	*Ha*-***0****P*	5.73±0.38**b**	196.33±26.08**b**	24.89±16.81**a**	204.33±122.09**a**	2.15±0.21**bc**	0.86±0.19**a**	22.40±2.62**b**	41.03±4.97**ab**
	Hb-***0****P*	7.23±0.31**a**	234.33±18.50**a**	30.77±2.77**a**	262.33±12.58**a**	2.77±0.22**a**	0.61±0.05**b**	27.53±0.42**a**	47.53±5.49**a**
	Hc-***0****P*	5.97±0.32**b**	195.33±2.31**b**	2.51±1.58**b**	253.00±10.15**a**	2.48±0.38**ab**	0.66±0.16**ab**	27.13±0.98**a**	42.43±7.94**ab**
	Ly-***0****P*	5.50±0.17**b**	150.67±10.69**c**	5.03±4.74**b**	80.67±48.01**b**	1.96±0.16**c**	0.50±0.07**b**	25.63±2.95**ab**	36.07±2.65**b**
**Tillering stage (2P)**	*Ha*-***2****P*	5.73±0.15**b**	185.00±11.53**a**	5.69±2.85**a**	107.33±43.43**bc**	2.43±0.31**a**	1.00±0.08**a**	21.97±1.63**b**	44.70±1.78**a**
	Hb-***2****P*	6.70±0.46**a**	195.00±13.11**a**	19.27±11.71**a**	217.67±52.01**a**	2.52±0.27**a**	0.62±0.03**b**	29.17±1.10**a**	46.57±2.96**a**
	Hc-***2****P*	5.70±0.10**b**	195.67±10.60**a**	8.77±12.49**a**	178.00±40.15**ab**	2.68±0.44**a**	0.73±0.29**ab**	29.70±2.08**a**	45.00±5.36**a**
	Ly-***2****P*	5.13±0.21**c**	149.33±27.61**b**	5.35±4.77**a**	42.67±8.02**c**	1.78±0.11**b**	0.46±0.09**b**	23.90±1.25**b**	36.30±2.86**b**
**Heading stage (3P)**	*Ha*-***3****P*	5.20±0.06**b**	163.67±17.24**ab**	17.37±7.35**a**	171.33±36.75**a**	2.25±0.07**b**	0.72±0.04**a**	18.83±1.48**c**	42.07±1.79**a**
	Hb-***3****P*	5.99±0.29**a**	171.33±18.56**a**	17.23±9.72**a**	228.33±58.38**a**	2.26±0.02**b**	0.64±0.08**a**	25.93±1.36**a**	43.00±2.81**a**
	Hc-***3****P*	4.97±0.14**b**	182.67±17.50**a**	13.66±16.58**a**	184.00±23.52**a**	2.65±0.20**a**	0.81±0.39**a**	22.40±1.14**b**	43.97±6.71**a**
	Ly-***3****P*	5.04±0.10**b**	131.67±25.15**b**	5.61±9.43**a**	42.67±8.50**b**	1.73±0.34**c**	0.45±0.05**a**	24.20±1.15**b**	27.23±4.40**b**
**Ripening stage (4P)**	*Ha*-***4****P*	6.83±0.10**a**	165.67±17.01**ab**	16.00±5.04**a**	162.67±26.50**b**	2.14±0.16**ab**	0.72±0.06**a**	23.80±2.76**b**	40.00±4.27**a**
	Hb-***4****P*	5.85±0.09**b**	156.33±10.26**ab**	19.27±7.11**a**	223.67±31.88**a**	2.56±0.46**a**	0.69±0.13**a**	34.43±2.00**a**	43.53±2.46**a**
	Hc-***4****P*	5.51±0.21**b**	175.00±7.94**a**	12.79±15.43**a**	167.67±15.04**b**	2.44±0.33**a**	0.77±0.31**a**	31.23±2.22**a**	38.77±5.49**a**
	Ly-***4****P*	5.53±0.28**b**	140.00±20.66**b**	4.73±7.68**a**	36.33±6.66**c**	1.73±0.22**b**	0.45±0.06**a**	24.67±0.55**b**	29.77±4.53**b**

Table S4 Permutational Multivariate Analysis of Variance (Adonis) shows the effect of soil properties in the different stages of crop transplanting on yield of super rice with three methods. *P<0.05, **P<0.01.

**Adonis**	**Bray**		**Euclid**		**Horn**	
	R^2	Pr(>F)	R^2	Pr(>F)	R^2	Pr(>F)
**Before transplanting**	**0.584***	0.011	**0.556****	0.009	**0.705****	0.009
**Tillering stage**	**0.615****	0.002	**0.505****	0.007	**0.658****	0.004
**Heading stage**	**0.754****	0.002	**0.687****	0.005	**0.840****	0.004
**Ripening stage**	**0.756****	0.003	**0.723****	0.005	**0.839****	0.003

Table S5 Pearson positive correlations between crop yield and several soil physical and chemical properties during four stages. *P<0.05, **P<0.01. (AN: available nitrogen; AP: available phosphorus; AK: available potassium; TN: total nitrogen; TP: total phosphorus; TK: total potassium; SOM: soil organic matter.)

**Pearson correlation**	**yield**			
	Pre-transplanting stage	Tillering stage	Heading stage	Ripening stage
**pH**	0.421	**0.583***	0.248	0.367
**AN**	**0.715****	**0.782****	**0.728****	**0.667***
**AP**	0.343	0.232	0.416	0.478
**AK**	**0.758****	**0.708****	**0.845****	**0.868****
**TN**	0.560	**0.791****	**0.824****	**0.677***
**TP**	0.532	**0.603***	**0.586***	**0.646***
**TK**	0.018	0.391	-0.314	0.451
**SOM**	0.502	**0.784****	**0.884****	**0.732****

Table S6 Permutational Multivariate Analysis of Variance (Adonis) shows the effect of bacterial diversity on crop yield in four stages. *P<0.05, **P<0.01.

**Adonis**	**Bray**		**Euclid**		**Horn**	
	R^2	Pr(>F)	R^2	Pr(>F)	R^2	Pr(>F)
**Pre-transplanting stage**	**0.378***	0.031	**0.336***	0.044	**0.693***	0.028
**Tillering stage**	0.224	0.115	0.235	0.115	0.21	0.161
**Heading stage**	**0.492****	0.007	**0.518***	0.012	**0.646***	0.014
**Ripening stage**	0.069	0.426	0.082	0.378	-0.091	0.843

Table S7 Permutational Multivariate Analysis of Variance (Adonis) shows the effect of bacterial communities' structure in OUTs level on crop yield in four stages. *P<0.05, **P<0.01.

**Adonis**	**Bray**		**Euclid**		**Horn**	
	R^2	Pr(>F)	R^2	Pr(>F)	R^2	Pr(>F)
**Pre-transplanting stage**	0.129	0.128	0.186	0.063	0.134	0.21
**Tillering stage**	**0.172***	0.023	0.178	0.057	0.182	0.081
**Heading stage**	**0.178***	0.018	**0.300****	0.005	**0.347***	0.019
**Ripening stage**	0.129	0.103	0.106	0.288	0.100	0.339

Table S8 Permutational Multivariate Analysis of Variance (Adonis) shows the effect of bacterial communities' structure in genus level on crop yield in four stages. *P<0.05, **P<0.01.

**Adonis**	**Bray**		**Euclid**		**Horn**	
	R^2	Pr(>F)	R^2	Pr(>F)	R^2	Pr(>F)
**Pre-transplanting stage**	0.121	0.203	0.156	0.189	0.152	0.251
**Tillering stage**	0.169	0.087	0.183	0.158	0.214	0.190
**Heading stage**	**0.242***	0.013	**0.363****	0.028	0.423	0.083
**Ripening stage**	0.100	0.347	0.085	0.386	0.067	0.445

Table S9 Relative abundances (%) of the dominant bacterial phyla (a) and class (b) in soil at heading stage; the numerical value is the relative abundance of the sequence number of the bacteria taxa in each of the 48000 sequences.

**Classification**	**Heading stage**			
	Ha-3P	Hb-3P	Hc-3P	Ly-3P
(a) Phylum				
Proteobacteria	21.31	33.14	23.45	25.61
Unclassified	18.64	16.86	18.75	20.10
Acidobacteria	18.74	16.08	18.33	14.28
Chloroflexi	18.25	10.10	16.97	16.30
Verrucomicrobia	3.98	4.88	4.71	5.29
Bacteroidetes	2.20	5.69	2.31	2.23
Firmicutes	2.99	2.67	3.38	2.75
Euryarchaeota	3.59	1.65	2.22	2.54
Crenarchaeota	2.30	0.23	1.86	5.18
Planctomycetes	3.00	3.51	3.04	0.98
Others	5.00	5.21	4.98	4.74
(b) Class				
Unclassified	28.88	27.30	29.30	31.79
Deltaproteobacteria	7.20	13.15	8.53	9.79
Anaerolineae	11.35	6.41	11.30	9.29
Betaproteobacteria	5.30	6.74	5.25	6.49
Alphaproteobacteria	4.61	5.89	4.43	4.33
Subdivision3	3.17	3.94	3.73	3.66
Acidobacteria_Gp1	2.85	3.23	2.55	2.37
Acidobacteria_Gp18	3.62	2.83	4.26	3.28
Gammaproteobacteria	2.13	4.39	2.71	2.44
Acidobacteria_Gp6	3.24	2.34	3.64	2.00
Acidobacteria_Gp3	2.82	2.69	2.04	2.56
Planctomycetacia	2.68	3.33	2.79	0.84
Acidobacteria_Gp7	1.32	1.19	1.65	1.37
Sphingobacteria	0.69	1.55	0.62	0.83
Acidobacteria_Gp2	0.84	0.69	0.56	0.29
Spartobacteria	0.61	0.46	0.68	1.36
Actinobacteria	0.79	1.74	0.98	0.40
Acidobacteria_Gp4	1.37	0.32	0.52	0.68
Methanomicrobia	1.60	0.16	0.64	0.98
Acidobacteria_Gp16	0.77	0.39	0.65	0.41
Others	8.48	9.61	9.26	6.86

Table S10 Significantly Pearson correlations between microbial community and soil environmental factors at heading stage were shown, based on phylum level. *P<0.05, **P<0.01. (AK: available potassium; TN: total nitrogen; TK: total potassium; SOM: soil organic matter.)

**Heading stage**	**pH**	**TK**	**AN**	**AK**	**SOM**	**Yield**
**Proteobacteria**	**0.743****	**0.680***	-0.318	0.275	0.031	-0.036
**Unclassified**	**-0.594***	-0.122	0.007	-0.522	-0.300	-0.439
**Chloroflexi**	**-0.621***	**-0.655***	-0.072	-0.406	-0.071	-0.061
**Verrucomicrobia**	-0.055	0.567	-0.279	-0.285	**-0.604***	-0.550
**Bacteroidetes**	**0.752****	**0.591***	0.044	0.572	0.256	0.210
**Crenarchaeota**	-0.547	0.000	-0.222	**-0.666***	-0.455	**-0.647***
**Planctomycetes**	0.315	-0.129	**0.582***	0.573	0.451	**0.668***

Table S11 Significantly Pearson correlations between bacterial communities and soil environmental factors in heading stage were shown, based on genus level. *P<0.05, **P<0.01. (AN: available nitrogen; AK: available potassium; TN: total nitrogen; TP: total phosphorus; TK: total potassium; SOM: soil organic matter.)

Genus	pH	AN	AK	TN	TP	TK	SOM	Yield
Acidobacteria								
Gp6	-0.357	**0.577***	0.384	**0.692***	0.425	**-0.678***	0.570	**0.699***
Gp17	0.090	0.508	**0.592***	**0.682***	**0.589***	-0.294	0.550	**0.823****
Gp24	0.137	0.494	0.510	**0.756****	0.571	0.071	**0.590***	**0.679***
Gp25	-0.181	**0.748****	0.357	**0.611***	0.059	-0.099	0.397	**0.577***
Proteobacteria								
Geobacter	0.085	**-0.772****	-0.425	**-0.874****	**-0.617***	0.126	**-0.619***	**-0.667***
Syntrophorhabdus	0.403	-0.551	-0.360	-0.498	-0.341	**0.777****	-0.485	**-0.606***
Phaselicystis	-0.150	**-0.647***	**-0.621***	**-0.650***	-0.468	0.206	**-0.767****	**-0.680***
Thiobacter	-0.267	-0.548	-0.511	**-0.694***	-0.456	-0.072	**-0.715****	**-0.580***
Cystobacter	**0.743****	0.535	**0.735****	0.502	0.407	0.250	**0.607***	**0.707***
Chondromyces	0.293	**-0.733****	-0.430	**-0.619***	-0.150	0.397	-0.512	**-0.577***
Verrucomicrobia								
Spartobacteria-sedis	-0.417	-0.530	**-0.682***	-0.497	-0.284	0.112	**-0.769****	**-0.690***
Chloroflexi								
Longilinea	0.428	0.173	0.411	0.324	0.249	-0.305	0.561	**0.578***
OD1								
OD1-sedis	-0.504	-0.563	**-0.856****	**-0.660***	-0.440	0.076	**-0.754****	**-0.872****
Planctomycetes								
Blastopirellula	-0.064	0.388	0.507	0.447	0.406	**-0.623***	0.485	**0.698***
Armatimonadetes								
Armatimonadetes-gp2	0.014	**0.783****	0.425	0.517	0.112	-0.403	0.560	**0.603***
Armatimonadetes-gp5	-0.194	-0.477	-0.574	**-0.706***	-0.489	-0.099	**-0.759****	**-0.661***
Firmicutes								
Pasteuria	0.050	0.207	0.497	**0.642***	**0.766****	-0.016	0.477	**0.590***
**Yield**	0.248	**0.728****	**0.845****	**0.824****	**0.586***	-0.314	**0.884****	1.000
